# Type 1 Diabetes Mellitus in a Child With Genetically Confirmed Alström Syndrome: An Unusual Autoimmune Phenotype

**DOI:** 10.7759/cureus.109440

**Published:** 2026-05-22

**Authors:** Ali S Alquraishi, Nada Al Alammar, Musa M Saad, Ruba A Alqahtani

**Affiliations:** 1 Department of Pediatrics, Endocrinology Unit, Armed Forces Hospital Southern Region, Khamis Mushait, SAU; 2 Department of Pediatrics, Armed Forces Hospital Southern Region, Khamis Mushait, SAU; 3 Department of Day Surgery, Armed Forces Hospital Southern Region, Khamis Mushait, SAU

**Keywords:** alms1 mutation, alström syndrome, autoimmune diabetes, c-peptide, type 1 diabetes mellitus (t1dm)

## Abstract

Alström syndrome (AS) is a rare autosomal recessive multisystem disorder caused by pathogenic variants in the *ALMS1* gene and is classically associated with obesity, insulin resistance, and type 2 diabetes mellitus (T2DM). We report a 9-year-and-10-month-old girl with genetically confirmed Alström syndrome who developed diabetes mellitus at 5 years of age. Unlike the typical metabolic phenotype reported in Alström syndrome, the patient demonstrated positive anti-glutamic acid decarboxylase (GAD) and insulinoma-associated antigen-2 (IA-2) antibodies, progressive decline in endogenous insulin secretion, and persistent insulin dependence, consistent with autoimmune type 1 diabetes mellitus (T1DM). Despite obesity, there were no significant clinical features of severe insulin resistance. Glycemic control progressively deteriorated over follow-up despite intensive insulin therapy. This case highlights the phenotypic variability of diabetes in Alström syndrome and emphasizes the importance of comprehensive endocrine evaluation to ensure accurate diabetes classification and appropriate management.

## Introduction

Alström syndrome (AS) is a rare inherited ciliopathy characterized by progressive multiorgan dysfunction involving the visual, auditory, endocrine, cardiac, hepatic, and renal systems [1]. Alström syndrome (AS; OMIM 203800) is a rare autosomal recessive multisystem disorder caused by pathogenic variants in the *ALMS1* gene [2]. The estimated prevalence is less than 1 per 100,000 population [2]. The disorder typically presents during childhood with cone-rod retinal dystrophy, photophobia, nystagmus, obesity, and progressive metabolic dysfunction, while additional manifestations, including sensorineural hearing loss, cardiomyopathy, hepatic disease, and renal impairment, may evolve gradually with age [3]. Clinical manifestations are highly variable, often leading to delayed diagnosis and underrecognition of the condition.

Metabolic dysfunction is a major component of AS and typically develops early in life. Hyperinsulinemia and severe insulin resistance are considered hallmark endocrine features, frequently progressing to type 2 diabetes mellitus (T2DM) during adolescence or early adulthood [4]. Many affected patients demonstrate obesity, acanthosis nigricans, hypertriglyceridemia, and fatty liver disease [4]. Several reports have described markedly elevated insulin requirements and clinical improvement with insulin-sensitizing agents such as metformin, supporting the predominance of insulin resistance in the pathophysiology of diabetes associated with AS [5,6].

In most reported cases, endogenous insulin secretion is initially preserved, pancreatic autoantibodies are negative, and the diabetes phenotype resembles insulin-resistant T2DM rather than autoimmune beta-cell destruction [7].

Although diabetes mellitus is common in AS, autoimmune diabetes has rarely been described in association with the syndrome. Most published reports document biochemical and clinical profiles consistent with insulin-resistant diabetes rather than type 1 diabetes mellitus (T1DM) [7,8]. Therefore, the occurrence of positive pancreatic autoantibodies together with progressive beta-cell failure in a patient with genetically confirmed AS represents an unusual and clinically important phenotype that may complicate diabetes classification and management.

Here, we report a child with genetically confirmed Alström syndrome who developed diabetes mellitus associated with positive pancreatic autoantibodies and progressive decline in C-peptide levels, indicating severe beta-cell failure consistent with a T1DM phenotype. This case highlights an unusual endocrine presentation of AS and emphasizes the importance of comprehensive diabetes classification in syndromic patients.

## Case presentation

A 9-year-and-10-month-old Saudi girl with genetically confirmed Alström syndrome was evaluated for diabetes mellitus initially diagnosed at the age of 5 years. The diagnosis of Alström syndrome had been established at 3 years of age following molecular genetic testing, which identified a homozygous pathogenic variant in the *ALMS1* gene (NM_015120: exon 19: c.11870-2A>T), a known Saudi mutation inherited in an autosomal recessive pattern. Family history was significant for an affected younger sister with genetically confirmed Alström syndrome and a 17-year-old brother with insulin-dependent diabetes mellitus managed with an insulin pump, reportedly diagnosed during childhood. The patient’s mother had hypothyroidism treated with levothyroxine. There was no confirmed family history of other autoimmune endocrine disorders. Parental consanguinity was reported.

The patient initially presented with polyuria and polydipsia without associated weight loss, ketosis, or diabetic ketoacidosis (DKA). Diabetes management required initiation of insulin therapy from diagnosis, and the patient remained persistently insulin-dependent throughout follow-up. At the most recent assessment, her weight was 60 kg (>97th percentile), and her height was 137 cm (approximately 50th percentile), corresponding to a body mass index (BMI) of 32 kg/m² (>97th percentile), consistent with obesity. Progressive weight gain had been documented during longitudinal follow-up after diabetes diagnosis. Pubertal status was not formally documented at the time of evaluation. Physical examination revealed no evidence of acanthosis nigricans either at presentation or during subsequent endocrine follow-up visits, and there were no other clinical signs suggestive of marked insulin resistance.

**Figure 1:**
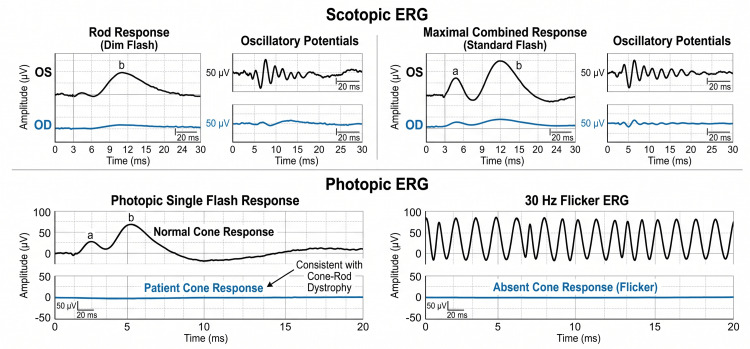
Representative ERG findings demonstrating cone-rod dystrophy in Alström syndrome Representative schematic illustration of ERG findings based on the patient’s documented ophthalmologic evaluation and created for educational visualization purposes. The figure is not an original patient-derived ERG tracing. Scotopic and photopic ERG patterns demonstrate markedly reduced and absent cone responses consistent with cone-rod dystrophy characteristic of retinal involvement in Alström syndrome. ERG: electroretinography

Ophthalmologic evaluation demonstrated cone-rod dystrophy with absent cone responses on scotopic electroretinography, associated with nystagmus since infancy and persistent photophobia. Representative electroretinography findings illustrating severe cone dysfunction consistent with cone-rod dystrophy are presented in Figure 1.

Audiologic assessment was normal, with no evidence of sensorineural hearing loss at the time of evaluation. Cardiac assessment revealed situs solitus with levocardia, mild tricuspid regurgitation, and trace mitral regurgitation, without clinically significant structural cardiac abnormalities.

**Figure 2:**
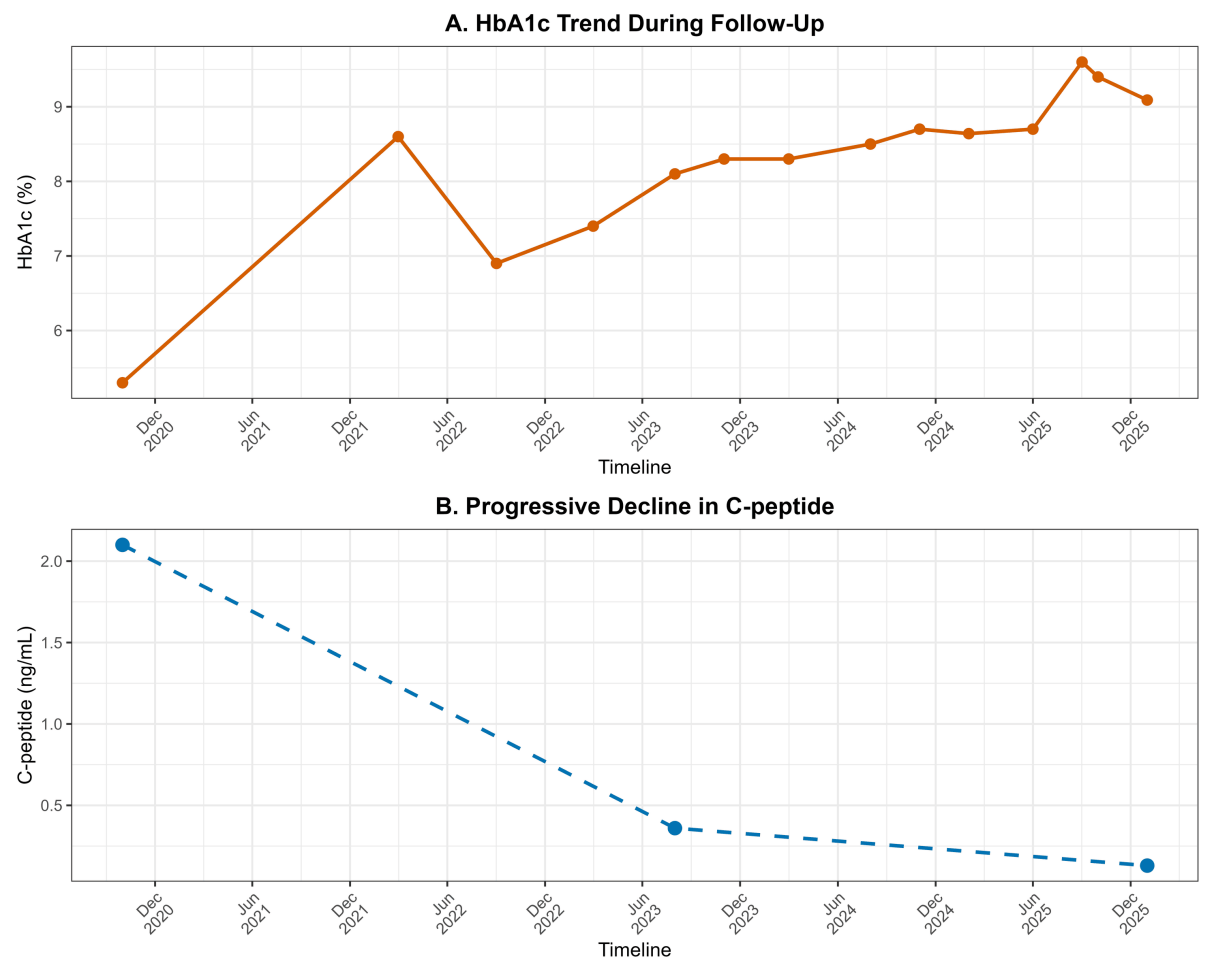
Longitudinal trends of glycemic control and endogenous insulin secretion in a child with genetically confirmed Alström syndrome and autoimmune diabetes Longitudinal follow-up from October 2020 to January 2026 demonstrating progressive deterioration of glycemic control and decline in endogenous insulin secretion despite intensive insulin therapy. (A) Serial HbA1c measurements showed persistent worsening of glycemic control during endocrine follow-up. (B) Serial C-peptide measurements obtained at selected time points during follow-up demonstrated progressive decline from 2.1 ng/mL in 2020 to 0.13 ng/mL in 2026, consistent with progressive autoimmune beta-cell failure and persistent insulin dependence. HbA1c: hemoglobin A1c

Laboratory investigations were obtained during serial endocrine evaluations between 2020 and 2026, with the most recent measurements corresponding to the latest clinical follow-up unless otherwise specified. Laboratory evaluation demonstrated evidence of autoimmune diabetes. Pancreatic autoantibodies were positive for anti-glutamic acid decarboxylase (anti-GAD) antibodies at 15.3 U/mL and insulinoma-associated antigen-2 (IA-2) antibodies at 1.80 U/mL, while zinc transporter 8 (ZnT8) antibodies were negative (<10 RU/mL). Initial fasting insulin and C-peptide levels were within normal ranges at 14.7 µIU/mL and 2.1 ng/mL, respectively. However, serial measurements demonstrated progressive decline in endogenous insulin secretion, with subsequent C-peptide levels decreasing to 0.36 ng/mL and eventually to 0.13 ng/mL, indicating marked beta-cell failure. The longitudinal progression of glycemic control deterioration and decline in endogenous insulin secretion during follow-up is illustrated in Figure 2.

Glycemic control progressively deteriorated over time despite insulin therapy. Hemoglobin A1c (HbA1c) increased from 5.3% in October 2020 to 9.09% in January 2026, with persistently elevated values during follow-up. Random blood glucose at the latest assessment was 13.07 mmol/L. Lipid profile demonstrated mildly elevated total cholesterol (5.21 mmol/L) and low-density lipoprotein (LDL) cholesterol (3.82 mmol/L), while triglyceride and high-density lipoprotein (HDL) cholesterol levels remained within acceptable ranges. Liver function tests, renal profile, and bone profile remained within normal limits throughout follow-up. A comprehensive summary of laboratory investigations with corresponding reference ranges is presented in Table 1.

**Table 1:** Summary of laboratory investigations and reference ranges Reference ranges are laboratory-specific and may vary slightly according to institutional standards. GAD: glutamic acid decarboxylase, IA-2: insulinoma-associated antigen-2, ZnT8: zinc transporter 8, HbA1c: hemoglobin A1c, HDL: high-density lipoprotein, LDL: low-density lipoprotein

Parameter	Date	Result	Unit	Reference range
Anti-GAD antibodies	2025	15.3	U/mL	Negative
IA-2 antibodies	2025	1.80	U/mL	<0.8
ZnT8 antibodies	2025	<10	RU/mL	<15
Fasting insulin	2020	14.7	µIU/mL	2-25
Initial C-peptide	2020	2.1	ng/mL	0.8-3.5
Subsequent C-peptide	2023	0.36	ng/mL	0.8-3.5
Most recent C-peptide	2026	0.13	ng/mL	0.8-3.5
Random blood glucose	2026	13.07	mmol/L	3.9-7.8
Latest HbA1c	2026	9.09	%	<5.7
Total cholesterol	2026	5.21	mmol/L	<5.2
Triglycerides	2026	1.11	mmol/L	<1.7
HDL cholesterol	2026	1.13	mmol/L	>1.0
LDL cholesterol	2026	3.82	mmol/L	<3.4

The patient was managed with a basal-bolus insulin regimen consisting of insulin degludec (Tresiba) and insulin aspart (NovoRapid). Basal insulin therapy consisted of 26 units administered nightly at 8 PM. Mealtime insulin dosing was adjusted according to an insulin-to-carbohydrate ratio of 1:25 with a correction factor of 1:50. Exact total daily insulin dose and bolus insulin requirements varied during follow-up and were not consistently documented. Despite ongoing endocrinology follow-up and multiple insulin dose adjustments, glycemic control remained suboptimal. Throughout follow-up, there was no history of diabetic ketoacidosis, severe hypoglycemia requiring hospitalization, or evidence of chronic microvascular complications.

Overall, this patient’s clinical course was notable for positive pancreatic autoantibodies, progressive decline in C-peptide levels, persistent insulin dependence, and absence of overt clinical features of severe insulin resistance. These findings were more consistent with an autoimmune type 1 diabetes mellitus phenotype rather than the insulin-resistant type 2 diabetes phenotype classically associated with Alström syndrome.

## Discussion

Alström syndrome is a rare ciliopathy caused by pathogenic variants in the *ALMS1* gene and is characterized by progressive multisystem involvement, including retinal degeneration, sensorineural hearing loss, obesity, insulin resistance, cardiomyopathy, and renal and hepatic dysfunction. According to Marshall et al. (2005), significant phenotypic variability exists among affected individuals, contributing to delayed recognition and diagnostic challenges [1]. Diabetes mellitus represents one of the major endocrine manifestations of the syndrome and is classically described as severe insulin-resistant diabetes resembling type 2 diabetes mellitus (T2DM) [6].

Previous studies consistently demonstrated that glucose dysregulation in Alström syndrome is predominantly driven by hyperinsulinemia and insulin resistance. According to Satman et al. (2002), affected patients often progress from obesity and hyperinsulinemia to impaired glucose tolerance and overt diabetes with gradual beta-cell dysfunction over time [7]. Similarly, Bettini et al. (2012) proposed that both insulin resistance and progressive beta-cell failure contribute to diabetes development in Alström syndrome, although insulin resistance remains the dominant metabolic abnormality [9].

Several published case reports further support the typical insulin-resistant phenotype associated with Alström syndrome. According to Radi et al. (2022), a young adult with genetically confirmed Alström syndrome presented with severe insulin resistance, acanthosis nigricans, markedly elevated insulin requirements, negative pancreatic autoantibodies, and preserved C-peptide levels [6]. The patient demonstrated clinical improvement after initiation of metformin and insulin-sensitizing therapy. Likewise, Ucan et al. (2017) described a patient with poorly controlled diabetes requiring extremely high insulin doses who subsequently achieved improved glycemic control after addition of metformin, emphasizing the central role of insulin resistance in the syndrome [5].

In contrast, our patient demonstrated several findings more consistent with autoimmune type 1 diabetes mellitus (T1DM) rather than the expected insulin-resistant phenotype. She had positive anti-GAD and IA-2 antibodies, a progressive decline in C-peptide levels from 2.1 ng/mL to 0.13 ng/mL, and persistent insulin dependence. Furthermore, despite obesity, she lacked clinical evidence of marked insulin resistance such as acanthosis nigricans or excessive insulin requirements. The progressive deterioration in glycemic control despite insulin therapy also supports ongoing autoimmune beta-cell destruction [10]. The distinguishing clinical, immunological, and metabolic features between the typical diabetes phenotype reported in Alström syndrome and the findings observed in our patient are summarized in Table 2.

**Table 2:** Clinical, immunological, and metabolic features supporting type 1 diabetes phenotype in the present patient compared with typical diabetes in Alström syndrome GAD: glutamic acid decarboxylase, IA-2: insulinoma-associated antigen-2, DKA: diabetic ketoacidosis

Feature	Typical diabetes in Alström syndrome	Present case
Usual diabetes phenotype	Type 2 diabetes mellitus	Type 1 diabetes mellitus phenotype
Pathophysiology	Severe insulin resistance	Autoimmune beta-cell failure
Obesity	Common	Present
Acanthosis nigricans	Common	Absent
Pancreatic autoantibodies	Usually negative	GAD and IA-2 positive
C-peptide at diagnosis	Preserved/high	Initially preserved
C-peptide over time	Gradual decline	Marked progressive decline (2.1 → 0.13 ng/mL)
Insulin resistance	Prominent	Minimal clinical evidence
Insulin requirement	Often high	Standard basal-bolus regimen
Response to metformin	Often beneficial	Not characteristic
DKA at presentation	Variable	Absent
Glycemic progression	Insulin resistance dominant	Progressive autoimmune insulin deficiency

Only limited reports have described atypical diabetes phenotypes in Alström syndrome. According to Bakar et al. (2017), a Saudi girl with Alström syndrome presented with diabetic ketoacidosis and weakly positive anti-insulin antibodies, described as “double diabetes” [4]. However, unlike our patient, preserved features of insulin resistance were still prominent. To our knowledge, reports documenting clear autoimmune diabetes characterized by positive pancreatic autoantibodies and severe progressive beta-cell failure in genetically confirmed Alström syndrome remain exceptionally rare.

The coexistence of autoimmune diabetes in this patient may reflect phenotypic variability, coincidental coexistence of type 1 diabetes mellitus independent of Alström syndrome, or a potential interaction between genetic and immune-mediated mechanisms [11]. An alternative consideration is a “double diabetes” phenotype, in which autoimmune beta-cell destruction coexists with obesity-related insulin resistance. However, our patient lacked prominent clinical features of severe insulin resistance, including acanthosis nigricans and markedly elevated insulin requirements, which are commonly reported in classical Alström-associated diabetes according to Radi et al. (2022) [6].

Although transient pancreatic autoantibody positivity or assay-related cross-reactivity may occasionally occur, the persistence of positive GAD and IA-2 antibodies together with progressive decline in C-peptide levels and sustained insulin dependence strongly supports true autoimmune beta-cell failure rather than nonspecific antibody positivity. Multiple pancreatic autoantibodies are generally associated with a higher likelihood of autoimmune diabetes progression [12]. Human leukocyte antigen (HLA) typing was not performed in our patient, which represents a limitation because diabetes-associated HLA susceptibility haplotypes could have provided additional support for autoimmune diabetes predisposition. Similarly, although the family history was notable for maternal hypothyroidism, there was no confirmed family history of other autoimmune endocrine disorders. This case highlights the importance of comprehensive endocrine evaluation in syndromic patients with diabetes. The presence of obesity or a syndromic diagnosis should not automatically lead to classification as type 2 diabetes mellitus without assessment of pancreatic autoantibodies and endogenous insulin secretion. Accurate diabetes classification is essential because it directly influences therapeutic decisions, monitoring strategies, and long-term prognosis.

## Conclusions

This case highlights an unusual presentation of diabetes in a child with genetically confirmed Alström syndrome, where the clinical, immunological, and biochemical findings were consistent with type 1 diabetes mellitus rather than the classically reported insulin-resistant type 2 diabetes phenotype. Positive pancreatic autoantibodies and progressive decline in C-peptide levels strongly supported autoimmune beta-cell destruction. This report emphasizes the marked phenotypic variability of Alström syndrome and the importance of comprehensive diabetes evaluation, including pancreatic autoantibodies and endogenous insulin secretion assessment, in syndromic patients. Early and accurate diabetes classification is essential to optimize treatment, guide long-term follow-up, and improve clinical outcomes. Clinicians should remain aware that autoimmune diabetes may rarely occur in patients with Alström syndrome despite the classically described insulin-resistant phenotype.
